# A Critical View over the Newest Antidiabetic Molecules in Light of Efficacy—A Systematic Review and Meta-Analysis

**DOI:** 10.3390/ijms24119760

**Published:** 2023-06-05

**Authors:** Teodor Salmen, Liviu-Ionut Serbanoiu, Ioana-Cristina Bica, Cristian Serafinceanu, Emir Muzurović, Andrej Janez, Stefan Busnatu, Maciej Banach, Ali Abbas Rizvi, Manfredi Rizzo, Anca Pantea Stoian

**Affiliations:** 1 Doctoral School, “Carol Davila” University of Medicine and Pharmacy, 020021 Bucharest, Romania; teodor.salmen@drd.umfcd.ro (T.S.); liviu-ionut.serbanoiu@drd.umfcd.ro (L.-I.S.); 2 Department of Diabetes, Nutrition and Metabolic Diseases, “Carol Davila” University of Medicine and Pharmacy, 020021 Bucharest, Romaniaanca.stoian@umfcd.ro (A.P.S.); 3 Department of Internal Medicine, Endocrinology Section, Clinical Center of Montenegro, Ljubljanska, 81000 Podgorica, Montenegro; dremir@t-com.me; 4 Faculty of Medicine, University of Montenegro, Kruševac bb, 81000 Podgorica, Montenegro; 5 Department of Endocrinology, Diabetes and Metabolic Diseases, University Medical Centre Ljubljana, University of Ljubljana, 1000 Ljubljana, Slovenia; andrej.janez@kclj.si; 6 Cardiology Department, Carol Davila University of Medicine and Pharmacy, 050474 Bucharest, Romania; stefan.busnatu@umfcd.ro; 7 Department of Preventive Cardiology and Lipidology, Medical University of Lodz, 93-338 Lodz, Poland; maciej.banach@icloud.com; 8 Department of Medicine, University of Central Florida College of Medicine, Orlando, FL 32827, USA; ali.rizvi@ucf.edu; 9 Department of Health Promotion, Mother and Child Care, Internal Medicine and Medical Specialties, University of Palermo, 90100 Palermo, Italy; manfredi.rizzo@unipa.it

**Keywords:** efficacy, novel antidiabetic noninsulinic drugs, HbA1c

## Abstract

The increase in life expectancy without a decrease in the years lived without disability leads to the rise of the population aged over 65 years prone to polypharmacy. The novel antidiabetic drugs can improve this global therapeutic and health problem in patients with diabetes mellitus (DM). We aimed to establish the efficacy (A1c hemoglobin reduction) and safety of the newest antidiabetic drugs (considered so due to their novelty in medical practice use), specifically DPP-4i, SGLT-2i, GLP-1 Ra, and tirzepatide. The present meta-analysis followed the protocol registered at Prospero with the CRD42022330442 registration number. The reduction in HbA1c in the DPP4-i class for tenegliptin was 95% CI −0.54 [−1.1, 0.01], *p* = 0.06; in the SGLT2-iclass for ipragliflozin 95% CI −0.2 [−0.87, 0.47], *p* = 0.55; and for tofogliflozin 95% CI 3.13 [−12.02, 18.28], *p* = 0.69, while for tirzepatide it was 0.15, 95% CI [−0.50, 0.80] (*p* = 0.65). The guidelines for treatment in type 2 DM are provided from cardiovascular outcome trials that report mainly major adverse cardiovascular events and data about efficacy. The newest antidiabetic non-insulinic drugs are reported to be efficient in lowering HbA1c, but this effect depends between classes, molecules, or patients’ age. The newest antidiabetic drugs are proven to be efficient molecules in terms of HbA1c decrease, weight reduction, and safety, but more studies are needed in order to characterize exactly their efficacy and safety profiles.

## 1. Introduction

The World Health Organization (WHO) reports an increase in life expectancy secondary to decreased mortality rates, but with no decrease in years lived without disability [1,2].

A concomitant rise in the population aged over 65 years, along with the non-transmittable chronic disease epidemics, including type 2 diabetes mellitus (T2DM), cardiovascular diseases (CVD), cancers, and chronic pulmonary diseases, describe a population group mainly affected by an association of various chronic diseases that require multiple medications in their therapeutic management [3,4,5]. Polypharmacy is a generally positive trend, despite its variable definition. It is accepted as being the administration of more than five drugs [6,7,8,9,10,11] and comes with negative effects, such as renal or hepatic function impairment [12,13], living disability, frailty [10,14], hemorrhagic and thrombo-embolic risk, malnutrition [15], long-term care placement or hospitalization [10,16,17], decreased quality of life, or mortality [5,6,7,10,18,19,20]; that came in opposition to the benefits of the antidiabetic, cardioprotective molecules that are proven efficient, safe, and non-inferior to the previous antidiabetic drugs. However, the main challenge is represented by the requirement to establish a proper and predictable treatment [21] and simultaneously avoid poor adherence in self-administering multiple drugs [22], so emphasis should be addressed on the need for a strict and periodic assessment of the risk-benefit balance when continuing or introducing a new therapeutic drug and promoting the personalized medicine [23,24,25]. 

T2DM is part of the cardio-renal and metabolic syndrome. It is not characterized only by a hyperglycemic status but also by the concomitant complications that appear, both microvascular complications, respectively, retinopathy, neuropathy, or nephropathy; and macrovascular complications, respectively, CVD with its components like stroke, myocardial infarction (MI), coronary artery disease, and peripheral arterial disease [26,27]. Despite the associated comorbidities, other risk factors for polypharmacy in patients with T2DM are represented by older age, that when associated with cognitive impairment may transform polypharmacy into a genuine burden; medication side effects or direct-to-consumer advertisements that can favor the patients to start administering drugs for erectile dysfunction or restless leg syndrome, that are not primarily needed [27]. Therefore, it is important to have comprehensive management to minimize its possible negative consequences [27].

In T2DM, in acute conditions, such as acute MI, stroke, or congestive heart failure, insulin is the main indicated treatment; otherwise, the recommended therapeutic approach includes antidiabetic non-insulin drugs. The novel antidiabetic non-insulin drugs are represented by dipeptidyl peptidase-4 inhibitor (DPP-4i), sodium glucose-2 transporter inhibitors (SGLT-2i), glucagon-like peptide one receptor agonists (GLP-1 Ra), and a dual glucose-dependent insulinotropic polypeptide–GLP-1 receptor agonist that is represented by tirzepatide. Their benefits are secondary to their pleiotropic effects, which exceed only an improvement in the glycemic control, reflected by an improved level of A1C hemoglobin or blood glucose, respectively, due to a reduction in the patient’s body weight, improvement of the lipidic profile, blood pressure (BP) both systolic and diastolic, along with pleiotropic effects, such as a decreased marker of inflammation [28,29,30]. Because T2DM is included in the cardio-renal metabolic syndrome, it is also important to emphasize the benefits of the novel antidiabetic non-insulin drugs from the large cardiovascular outcome trials (CVOT); specifically, a reduction in the major adverse cardiovascular events (MACE), including acute MI, stroke, CV mortality, all-cause mortality, and CV safety [28,29,30].

Aim of the study: The primary endpoint of the study is to establish if there are any differences in the efficacy profile (A1c hemoglobin reduction) for the newest antidiabetic drugs, respectively, DPP-4i, SGLT-2i, GLP-1 Ra, and tirzepatide. The secondary endpoint is to establish differences in the safety (frequency of adverse reaction (AR), such as hypoglycemia–mild (54–70 mg/dL) or severe (<36 mg/dL), and the frequency of class-specific AR profiles for the newest antidiabetic non-insulin drugs—DPP-4i, SGLT-2i, GLP-1 Ra, and tirzepatide.

## 2. Methods

We developed an easily reproducible protocol for our study following the recommendations of preferred reporting items for systematic reviews and meta-analyses (PRISMA) for the systematic review protocol checklist [31]. Furthermore, we used the population, intervention, comparison, outcome, and study design (PICOS) strategy to guide our study rationale and make a clear, systematic literature search that answers the question: “Is the use of the newest antidiabetic drugs efficient and safe for patients?”.

The studies will be included after searching in two databases, MEDLINE (using PubMed) and Web of Science, using the Boolean operators “AND” and “OR”, and the search strategy: “(efficacy OR safety) AND (novel antidiabetic drugs)”. The inclusion and exclusion criteria are as follows:

- Inclusion criteria: only experimental articles, both clinical trials and randomized controlled trials, published in full-text version in the last 10 years, that include human population over 18 years of age with T2DM, which are prescribed at least one class of novel non-insulin drugs, respectively, DPP-4i, SGLT-2i, GLP-1 Ra and tirzepatide;
- Exclusion criteria: abstracts, short communications, reviews, letters to editors, commentaries, or studies published in a language other than English, published more than 10 years ago, and studies on cell cultures or mammals.

**Figure 1:**
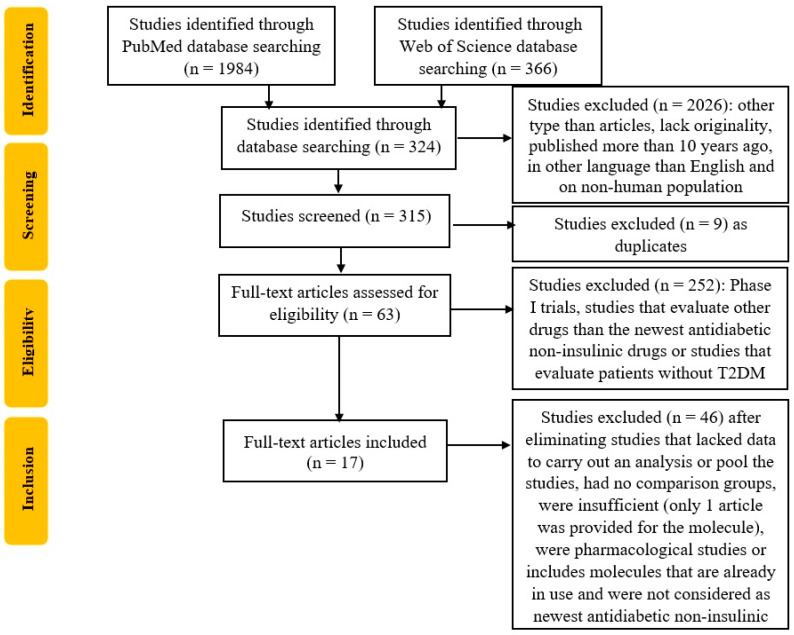
Flowchart of the study selection process according to PRISMA guidelines.

Furthermore, the duplicates will be eliminated, and two reviewers will analyze the articles for their relevance, as seen in the flow diagram in Figure 1. To improve the search, each list of selected titles will be shared, and a second screening of the previously selected titles will take place. Finally, a third researcher will be designated to arbitrate any problems that could appear in the selection process.

For data synthesis, we exclude studies with less than two of the same molecules, with insufficient or irrelevant data, or lacking originality. A random effect meta-analysis will combine risk and hazard ratios for individual studies.

Trial registration number: A protocol was submitted to Prospero-International prospective register of systematic reviews to obtain a registration number for our study, respectively, CRD42022330442.

## 3. Results and Discussions

For DPP-4i, the molecule with a sufficient number of studies [32,33,34,35,36], respectively five, was tenegliptin. The studies’ characteristics (study type, including population and its repartition in control and experimental groups, follow-up duration, mean age, mean HbA1c and mean glycemia, number of hypoglycemic events, and class-specific AR) are shown in Table 1. 

**Figure 2:**
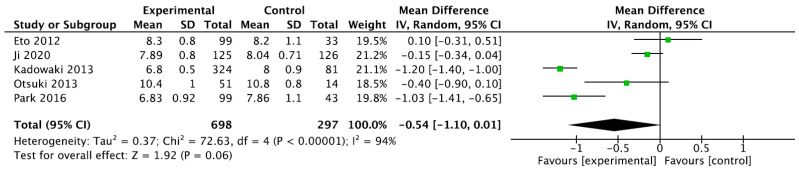
Forrest plot graphic representation of HbA1c for tenegliptin [32,33,34,35,36]. Green square represents the mean value for each study. Black square represents the mean value of the studies.

The results showed that teneligliptin is a promising antidiabetic drug in reducing blood sugar levels with statistical significance for HbA1c (95% CI −0.65 [−1.10, 0.01], *p* = 0.06), as seen in Figure 2.

For iSGLT-2, the molecules studied enough (five studies) were ipragliflozin [37,38,39,40,41] and tofogliflozin (two studies) [42,43], and the studies’ characteristics (study type, including population and its repartition in control and experimental groups, follow-up duration, mean age, mean HbA1c and mean glycemia, number of hypoglycemic events, and class-specific AR) are shown in Table 2. 

**Figure 3:**
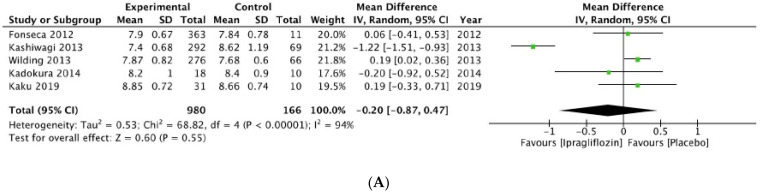

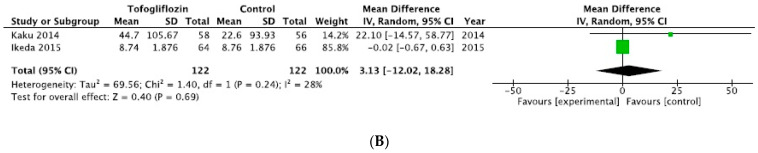
Forrest plot graphic representation of HbA1c for (**A**) ipragliflozin [37,38,39,40,41] and (**B**) tofogliflozin [42,43]. Green square represents the mean value for each study. Black square represents the mean value of the studies.

The results for ipragliflozin and tofogliflozin had no statistical significance, as seen in Figure 3A,B, respectively.

For dual glucose-dependent insulinotropic polypeptide–GLP-1 receptor agonist represented by tirzepatide [44,45,46,47,48], the studies’ characteristics (study type, including population and its repartition in control and experimental groups, follow-up duration, mean age, mean HbA1c and mean glycemia, number of hypoglycemic events and class-specific AR) are shown in Table 3.

**Figure 4:**
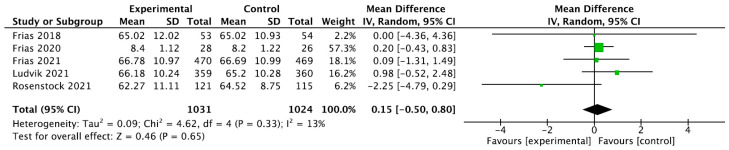
Forrest plot graphic representation of HbA1c for tirzepatide [44,45,46,47,48]. Green square represents the mean value for each study. Black square represents the mean value of the studies.

For tirzepatide, the 95% CI was 0.15 [−0.50, 0.80] (*p* = 0.65), as seen in Figure 4.

Data about each molecule’s weight reduction are shown in Table 4.

Our study provides comprehensive data about the efficacy of both novel and most recently developed non-insulin antidiabetic drugs. Our results confirm that these molecules are safe regarding glycemic control reflected by HbA1c and as shown by the limited AE or hypoglycemic events. The utility of this systematic review and meta-analysis is emphasized because the majority of data about this type of molecules are the results of large CVOTs, which are also the basis of the national and international guidelines for the treatment of T2DM [49], but in this type of studies, the primary outcomes are centered predominantly on MACE, such as CV death [48,49,50,51,52], nonfatal MI [49,50], nonfatal stroke [49,50], hospitalization, or urgent visits for heart failure (HF) [51,52,53] or sustained  ≥50% estimated glomerular filtration rate (eGFR) decline and end-stage kidney disease [54,55]. CVOTs also report data about efficacy, such as A1c hemoglobin [56,57,58], body weight loss [59,60,61], and systolic BP [57,58], and about safety eGFR rates [55] and the incidence of hypoglycemic episodes [58,62]. The benefit of the present study is the synthesis of these scattered results as a mean effect.

The limitation of the present study is the low number of included studies because there are molecules where only two studies met the inclusion criteria and, also, the scarce data about the molecules from each of the GIP-GLP1 and GIP-GLP-1-Glucagon that are not included.

### 3.1. DPP-4i

For the DPP-4i class, which is more known for its high tolerability, the metabolic control is reported to have a reduction in HbA1c of 0.6–0.8% [63]. There are also studies that reported that HbA1c reduction to be of only 0.3% in case of a longer follow-up period both for alogliptin and sitagliptin [64]. TECOS trial is a good example because in the first 4 months, sitagliptin obtained the largest decrease, and at 3 years, the benefit was only 0.3% [65], identical to the SAVOR-TIMI study [66] and similar to the CARMELINA trial where the reduction for linagliptin on HbA1c was only 0.36% [67]. At any rate, despite the modest decrease of HbA1c and the slight weight loss, compared to GLP-1 Ra, DPP-4i have few side effects, with a negligible risk of hypoglycemia [63].

From the AR point of view, a review by Gomez-Peralta et al. [63] reported that the incidence of hypoglycemic events is lower when comparing sitagliptin with a placebo or an insulin-increasing regimen; similar to vildagliptin as compared to a placebo, when adding a DPP-4i to an insufficient insulin regimen [63]. Moreover, it seems that even in the case of patients with T2DM and severe chronic kidney disease, the treatment with DPP-4i is safe regarding hypoglycemic events [63]. The data for the elderly population also show a safe profile for linagliptin compared to metformin, to sulfonylurea, to a basal insulin regimen, or to insulin treatment in general, while for vildagliptin, the evidence compares to insulin treatment [63]. Another important group that seems to benefit from DPP-4i, alone or when added to insulin regimens, are the hospitalized patients, where there is no increase in the hypoglycemic events but with better metabolic control [63].

### 3.2. SGLT-2i

In the case of SGLT-2i, in the DAPA-HF study, there is a reported reduction of HbA1c in the case of dapagliflozin of 0.21% [68], while other studies report a greater reduction of 0.89% [69], or even 2.66% but in those cases when the baseline HbA1c from the beginning of the study was higher [70]. In EMPEROR-Reduced (empagliflozin outcome trial in patients with chronic heart failure with reduced ejection fraction), empagliflozin is reported to reduce HbA1c, but without a specific value, so its range of efficacy on A1c hemoglobin is reported by other studies with the values that range between 0.11–0.82%, and similar to dapagliflozin a greater effect is encountered in the case of higher levels of A1c hemoglobin at the beginning of treatment [70,71].

Other efficacy elements are reported in the EMPAREG-OUTCOME trial, where empagliflozin reduces BP with 2.46 mmHg for systolic and 1.46 mmHg for diastolic, without raising the ventricular rhythm [70]. On the other hand, they are reported to be associated with a small increase in LDL-cholesterol and HDL-cholesterol levels and a small decrease in triglyceride and small dense LDL-cholesterol levels [70]. Moreover, empagliflozin reduced CV mortality in the EMPAREG-OUTCOME trial [72]. Empagliflozin is neutral on lipid metabolism and has a slower rate of decline in eGFR [51,73].

As AR, when talking about hypoglycemic events, empagliflozin, dapagliflozin, or canagliflozin does not increase their incidence [70], but it is important to emphasize that their use in association with representants of sulfonylurea favors the increase in hypoglycemic events incidence [70]. On the other hand, when talking about a class-specific AR, the urinary tract infections (UTIs) in large CVOTs or RCTs, the data are conflicting, from a decrease in incidence to no significant difference, and eventually to an increase in UTI incidence [70]. When taking each molecule, for dapagliflozin, the DECLARE-TIMI trial and real-life studies reported an increase in the incidence of UTIs, similar to empagliflozin in the EMPAREG-OUTCOME trial [70,74]. Another important class-specific AR is represented by genital infections, which are reported to have increased and even to favor the treatment discontinuation in the DECLARE-TIMI study, for dapagliflozin, in the EMPAREG-OTCOME trial for empagliflozin, and in the CANVAS trial for canagliflozin [70,74]. The cited factors reported to favor the occurrence of infections in the genital sphere are represented by the female gender and by the history of similar infections previously [70,74].

### 3.3. GLP-1 Ra

For the representants of the GLP-1 Ra class, semaglutide data are provided in the Pioneer trials. The reported HbA1c reduction ranged between 0.6% and 1.1% as compared to the placebo in the Pioneer 1 trial [75], of 1.3% as compared to the 0.9% reduction for empagliflozin [76], with the highest reported reduction of 1.7% in the Pioneer 9 trial [61]. The weight reduction ranged between 0.2 and 2.6 kg [75] compared to the placebo, or of 4.7 kg versus 3.8 kg in the case of empagliflozin [76]. The ARs reported no hypoglycemic events, including severe episodes, for oral semaglutide in the PIONEER 9 trial; one hypoglycemic event, without severe episodes, for oral semaglutide in the PIONEER 1 trial, and fewer hypoglycemic events than in the empagliflozin group, including one severe episode, for both oral semaglutide and empagliflozin, in the PIONEER 2 trial [61,75,76]. Moreover, when talking about the class-specific gastrointestinal (GI) AR, the most reported ARs were mainly constipation, along with a decrease of appetite, nausea, diarrhea, and abdominal discomfort for oral semaglutide in the PIONEER 9 trial; nausea and diarrhea, along with vomiting and decreased appetite for semaglutide in the PIONEER 1 trial; while nonserious nausea, of mild to moderate severity, but transient, along with diarrhea, vomiting, and a decreased appetite for semaglutide in the PIONEER 2 trial [61,75,76].

Discontinuation due to a drug-related AR was encountered in 10.7% of patients treated with oral semaglutide in the PIONEER 2 trial, for 7.4% of patients treated with oral semaglutide in the PIONEER 1 trial, and for 0.2% of patients treated with oral semaglutide in the PIONEER 9 trial [61,75,76].

Other trials that evaluate semaglutide are represented by Sustain trials, which reported a decrease of HbA1c of 1.55% (95% CI −1.74, −1.36), a body weight (BW) reduction of 3.73 kg (95% CI −4.54, −2.91) [77]. When talking about AR, there were no episodes of either hypoglycemia or severe hypoglycemia reported; and the class-specific GI AR encountered are nausea, vomiting, constipation, and diarrhea for semaglutide in sustain trials [77]. Regarding treatment discontinuation due to AR related to the treatment, it took place in only 3% of patients [77].

Exenatide was evaluated in duration trials and was reported to provide a reduction of HbA1c of 1.9% in weekly administration and a 1.5% reduction in case of daily administration in the Duration-1 trial [78]; a similar reduction in HbA1c of 1.5% in the weekly formulation was reported in the DURATION-2 trial [79]. On BW, both daily and weekly exenatide provide a reduction that ranges between 3.6 kg and 3.7 kg in Duration-1 [78], while in the DURATION-2 trial, there was only a 2.3 kg reduction in BW [79]. From the AR, no episodes of severe hypoglycemia, but a few episodes of minor hypoglycemia, especially in patients also treated with sulfonylurea, were encountered for exenatide in the DURATION-1 trial. In contrast, no episodes of severe hypoglycemia, but with 1% episode of minor hypoglycemia, were encountered for exenatide in the DURATION-2 trial [78,79]. The class-specific GI AR reported were nausea, vomiting, and diarrhea for exenatide in the DURATION-1 trial; and nausea, diarrhea, vomiting, and constipation for exenatide in the DURATION-2 trial [78,79]. It is important to emphasize that the GI AR were less likely to appear in the weekly presentation [78,79]. Discontinuation of treatment due to ARs was encountered for exenatide once a week in 6.1% of patients and, respectively, for exenatide twice a day in 4.8% of patients in the DURATION-1 trial, while 6.25% of patients discontinued the treatment due to related AR for exenatide once weekly in DURATION-2 trial [78,79].

Dulaglutide was evaluated in the AWARD trials, and for the metabolic control and AR for a follow-up of 26 weeks in the case of the AWARD-1 trial and 52 weeks in the case of the AWARD-2 trial. It was reported to provide a reduction of fasting plasma glucose of −43 ± 2 mg/dL and of −1.36 ± 0.08% for HbA1c in AWARD-1 trial [80] and of −27 ± 3 mg/dL for fasting plasma glucose level and of −1.08 ± 0.06% for HbA1c in the AWARD-2 trial [81]. The efficacy of BW reduction was −1.30 ± 0.29 kg for dulaglutide in the AWARD-1 trial [80] and −1.87 ± 0.24 kg for dulaglutide in the AWARD-2 trial [81]. From the AR perspective, hypoglycemic events were 10.4% for exenatide in the AWARD-1 trial, less than in the exenatide comparator, without episodes of severe hypoglycemia [80], while a rate of 55.3% hypoglycemic events as compared to 69.1% in the insulin glargine comparator group, with similar rated of severe hypoglycemic events (2 events in each group) for dulaglutide in the AWARD-2 trial [81]. The reported class-specific GI AR were nausea, diarrhea, and vomiting and were transient and mild to moderate in intensity for dulaglutide in the AWARD-1 trial [80]. Mild to moderate diarrhea and nausea were also reported in the AWARD-2 trial [81]. When discussing discontinuing the treatment due to developed AR, there were no significant differences between dulaglutide and comparators in the AWARD-1 and AWARD-2 trials [80,81]. 

### 3.4. Tirzepatide

The SURPASS clinical trials are the dedicated studies for tirzepatide that showed benefits on HbA1c reduction that vary from 1.24% to 2.58%, while the benefits on reduction of BW ranged from 5.4 kg to 11.7 kg [82]. ARs were specific to the GLP-1 Ra class and the GI AR—vomiting, nausea, constipation, and diarrhea, but with reduced CV events. It is important to emphasize that the discontinuation rate due to AR was low, at only 8.5%, and was not significantly higher when compared to the semaglutide comparator [82].

### 3.5. Future Perspectives

There are reported data that the innovation in the field of antidiabetic non-insulinic drugs is ongoing, and agents such as LY3437943, a novel triple agonist peptide at the glucagon receptor (GCGR), glucose-dependent insulinotropic polypeptide receptor (GIPR), and GLP-1R proved in phase 1 studies are safe and tolerable with similar benefits regarding weight and glycemic profiles to other incretins [83,84]; amylin analog represented by cagrilinitide that, without lifestyle intervention, reduced BW and improved glycemic control in healthy subjects [85]; cagrisema, a fixed combination of semaglutide and cagrilintide, that in phase 2 studies provided a higher HbA1c and BW reduction compared to both its components [86], while cotadutide bamadutide and SAR425899, dual GLP-1 and glucagon receptor agonists, reduced HbA1c along with BW in patients with T2DM and who are overweight or obese [87,88]. Other innovative treatments are represented by AMG-133, an antibody blocking the activation of glucose-dependent insulinotropic polypeptide receptor (GIPR) to which are conjugated GLP-1R peptide agonists that proved to have significant weight benefits in phase 1 studies on patients with obesity but without T2DM [89]. Also in development are molecules, such as a novel GLP-1R agonist, consisting of a DPP-IV-resistant GLP-1 fragment fused to the light chains of a humanized GLP-1R antibody by a peptide linker that acts like a structural highly specific antibody but with the properties of a GLP-1 agonist. It has proven efficient in normalizing glycemic fluctuations, improving β-cell function, and reducing BW in mice with T2DM [90,91]. Danuglipron, an oral GLP-1 Ra, also proved efficient and safe in treating mice with T2DM [92]. It is important to emphasize the importance of the association of nutritional intervention because there are reported data stating that intensive weight management, per se, can lead to important weight reduction and even to T2DM remission [93,94,95].

### 3.6. Strengths and Limitations

The major strength of our meta-analysis is that it showed that the newest antidiabetic non-insulinic drugs, respectively DPP-4i, SGLT-2i, GLP-1 Ra, and tirzepatide are proven to be efficient molecules in terms of HbA1c, weight reduction, and safety, such as hypoglycemia and a class-specific AR.

The primary limitation of our research is the predefined search, which limited the included studies and, respectively, the number of the molecules for each of the classes (e.g., only tenegliptin for DPP-4i, only ipragliflozin and tofogliflozin for SGLT-2i or not all the commercially available GLP-1 RA molecules) or the type populations that were available to be analyzed (e.g., AR comparison between elderly versus the rest of the population). Another limitation is the high rate of excluded full-text articles due to a high percentage of identified articles of Phase I trials, studies that evaluate drugs other than the newest antidiabetic non-insulinic drugs, or studies that evaluate patients without T2DM. Finally, another limitation is that drug dose, time, or disease severity were not evaluated.

## 4. Conclusions

There are few studies, including CVOTs, that report extensive data about the efficacy and safety of the novel non-insulin antidiabetic drugs, but they prove to be efficient molecules in terms of HbA1c and body weight reduction and offer these results in safe conditions, with low rates of hypoglycemic events, including severe ones; with low rates of specific AR and with low rates of discontinuation due to AR secondary to administered treatment. Moreover, there is hope for even better due to the innovative molecules still being developed. Nevertheless, there is a need for more studies of these novel non-insulin antidiabetic drugs, along with a need for translating the results into real-life settings to verify their favorable effects at the patient’s bedside.

## Figures and Tables

**Table 1:** Summarized characteristics of studies that included DPP4-i tenegliptin and its results of interest: drug name, patient’s background characteristics, study type, total population, experimental population, control population, HbA1c (hemoglobin A1c), the number of hypoglycemic events, and class-specific adverse reactions.

	Otsuki et al. [32]	Kadowaki et al. [33]	Eto et al. [34]	Hong et al. [35]	Ji et al. [36]
Drug	Teneligliptin				
Study type	RCT	RCT	RCT	RCT	RCT
Total population	51	324	99	142	254
Experimental population	14	81	33	99	125
Control population	29	80	32	43	126
Duration (weeks)	28	12	4	24	24
Age (years)	69.8 ± 8.5	57.5 ± 10.4	57.1 ± 8.7	NR	56 ± 10.2
Frequency of follow up	NR	NR	NR	NR	NR
HbA1c (%) [experimental]	10.4 ± 1	6.8 ± 0.5	8.3 ± 0.8	6.83 ± 0.92	NR
HbA1c (%) [control]	10.8 ± 0.8	8 ± 0.9	8.2 ± 1.1	7.86 ± 1.1	NR
*p*-value	*p* = 0.006				
Fasting plasma glucose (mg/dL) [experimental]	196 ± 59	125.3 ± 18.3	163.1 ± 30.8	135.2 ± 28.1	NR
Fasting plasma glucose (mg/dL) [control]	178 ± 89	148.2 ± 31.1	153.6 ± 31.9	161.2 ± 41.2	NR
Hypoglycemia (%)	0	3.7	0	0	3.1
Class-specific AR (%)	0	0	0	29.59	0.8

NR—not reported, RCT—randomized control trial, AR—adverse reaction.

**Table 2:** Summarized characteristics of studies that included SGLT2-ipragliflozin and tofogliflozin and their results of interest: drug name, patient’s background characteristics, study type, total population, experimental population, control population, HbA1c (hemoglobin A1c), and the number of hypoglycemic events and class-specific adverse reactions.

	Wilding et al. [37]	Fonseca et al. [38]	Kadokura et al. [39]	Kashiwagi et al. [40]	Kaku et al. [41]	Kaku et al. [42]	Ikeda et al. [43]
Drug	Ipragliflozin	Ipragliflozin	Ipragliflozin	Ipragliflozin	Ipragliflozin	Tofogliflozin	Tofogliflozin
Study type	RCT	RCT	RCT	RCT	RCT	RCT	RCT
Total population	343	1035	30	361	43	229	394
Experimental population	276	336	18	292	31	58	64
Control population	66	11	10	69	10	56	66
Duration (weeks)	12	12	2	12	NR	24	12
Age (years)	56.6 ± 8.9	54.2 ± 10.7	57.0 ± 13.19	56.0 ± 10.4	41.7 ± 14	56.6 ± 10.2	NR
Frequency of follow up	NS	4 weeks	1–14 days	NS	1–3 weeks	NS	NR
HbA1c (%) [experimental]	7.87 ± 0.82	7.90 ± 0.67	8.2 ± 1	7.4 ± 0.68	8.85 ± 0.72	8.34 ± 0.81	8.74 ± 1.876
HbA1c (%) [control]	7.68 ± 0.6	7.84 ± 0.78	8.4 ± 0.9	8.62 ± 1.19	8.66 ± 0.74	8.41 ± 0.78	8.76 ± 1.877
*p*-value	*p* = 0.55					*p* = 0.69	
Fasting plasma glucose (mg/dL) [Experimental]	154.8 ± 27	162.5 ± 44.8	177.5 ± 33.8	189.7 ± 36.7	150.9 ± 72.4	168.7 ± 29.6	NR
Fasting plasma glucose (mg/dL) [Control]	156.6 ± 37.8	160 ± 36.18	165.3 ± 35.1	130.6 ± 17.6	198.2 ± 75.4	168.8 ± 24.9	NR
Hypoglycemia (%)	2.8	1.5	0	1.4	70	1.7	0
Class-specific AR—UTI (%)	6.9	10.3	0	1.4	NR	1.7	4.5

NR—not reported, RCT—randomized control trial, AR—adverse reaction, UTI—urinary tract infections.

**Table 3:** Summarized characteristics of studies that included tirzepatide and their results of interest: drug name, patients’ background characteristics, study type, total population, experimental population, control population, HbA1c (hemoglobin A1c), and the number of hypoglycemic events and class-specific adverse reactions.

	Rosenstock et al. [44]	Frías et al. [45]	Ludvik et al. [46]	Frías et al. [47]	Frias et al. [48]
Drug	Tirzepatide				
Study type	RCT	RCT	RCT	RCT	RCT
Total population	478	1878	1444	318	111
Experimental population	115	470	360	53	29
Control population	121	469	359	51	26
Duration (weeks)	40	40	52	26	12
Age (years)	52.9 ± 12.3	55.9 ± 10.4	57.5 ± 10.2	56 ± 7.6	56.0 ± 10.13
Frequency of follow-up (weeks)	4	40	4	26	NR
HbA1c (%) [experimental]	7.85 ± 1.02	8.26 ± 1	4.81 ± 0.05	8.1 ± 1.1	8.4 ± 0.9
HbA1c (%) [control]	8.05 ± 0.8	8.25 ± 1.01	6.85 ± 0.05	8.1 ± 1	8.2 ± 1.22
*p*-value	*p* = 0.97				
Fasting plasma glucose (mg/dL) [experimental]	153.3 ± 40.4	172.4 ± 54.37	110.5 ± 1.9	164.8 ± 48.6	177.9 ± 54.68
Fasting plasma glucose (mg/dL) [control]	154.8 ± 40.3	171.4 ± 49.77	114.1 ± 1.8	178.1 ± 64.5	168.5 ± 62.06
Hypoglycemia (%)	7	1.7	2	0	17.9
Class specific AR—gastrointestinal (%)	41	44.9	NR	66	35.7

NR—not reported, RCT—randomized control trial, AR—adverse reaction.

**Table 4:** Weight reduction reported data for the molecules of interest.

Classes	Drugs	Studies	At Study End	At Baseline	*p* Value	Weight Parameter	Comparator
DPP-4i	Tenegliptin	Otsuki et al. [32]	57.1 ± 15.2	56.4 ± 14.5	0.08	BW (kg)	Ongoing antidiabetic therapy
		Kadowaki et al. [33]	95% CI 0.5 (0.1, 0.9)		<0.05	BW (kg)	Placebo
		Eto et al. [34]	24.8 ± 3.8	25.7 ± 4.5	0.282	BMI (kg/m^2^)	Placebo
		Hong et al. [35]	24.96 ± 2.51	25.07 ± 3.23	0.8436	BMI (kg/m^2^)	Placebo
		Ji et al. [36]	95% CI 0.22 (−0.32, 0.76)		0.4192	BW (kg)	Placebo
SGLT-2i	Ipragliflozin	Wilding et al. [37]	95% CI −2.21 (−2.83, 1.58)		<0.001	BW (kg)	Placebo
		Fonseca et al. [38]	95% CI −1.67 (−2.44, 0.89)		0.766	BW (kg)	Placebo
		Kadokura et al. [39]	−1.19 ± 0.44	75.07 ± 13.89	NR	BW (kg)	Placebo
		Kashiwagi et al. [40]	−2.1 ± 0.18		<0.001	BW (kg)	Placebo
		Kaku et al. [41]	−0.94 ± 0.89	66.53 ± 6.82	<0.001	BW (kg)	Placebo
	Tofogliflozin	Kaku et al. [42]	95% CI −2.971 (−3.440, −2.502)		<0.001	BW (kg)	Placebo
		Ikeda et al. [43]	95% CI −2.824 (−3.297, −2.351)		<0.0001	BW (kg)	Placebo
Dual GIP and GLP-1 Ra	Tirzepatide	Rosenstock et al. [44]	95% CI −8.8 (−10.3, −7.2)		<0.0001	BW (kg)	Placebo
		Frías et al. [45]	95% CI −6.2 (−7.1, −5.3)		<0.001	BW (kg)	Semaglutide 1 mg
		Ludvik et al. [46]	95% CI −15.2 (−16.2, −14.2)		<0.0001	BW (kg)	Insuline degludec
		Frías et al. [47]	−4.1 ± 0.31	32.2 ± 0.81	<0.01	BMI (kg/m^2^)	Placebo
		Frias et al. [48]	95% CI −5.2 (−7.5, −2.9)		<0.001	BW (kg)	Placebo

DPP-4i—dipeptidyl peptidase-4 inhibitor; SGLT-2i—sodium glucose-2 transporter inhibitors; GLP-1 Ra—glucagon-like peptide one receptor agonist; BW—body weight; CI—confidence interval; BMI—body mass index; NR—not reported.

## Data Availability

Not applicable.
